# Supplementary figures and images for: Different CprABC amino acid sequences affect nisin A susceptibility in Clostridioides difficile isolates
Source: PLoS One. 2023 Jan 20;18(1):e0280676. doi: 10.1371/journal.pone.0280676 (PMC9858009; doi:10.1371/journal.pone.0280676)

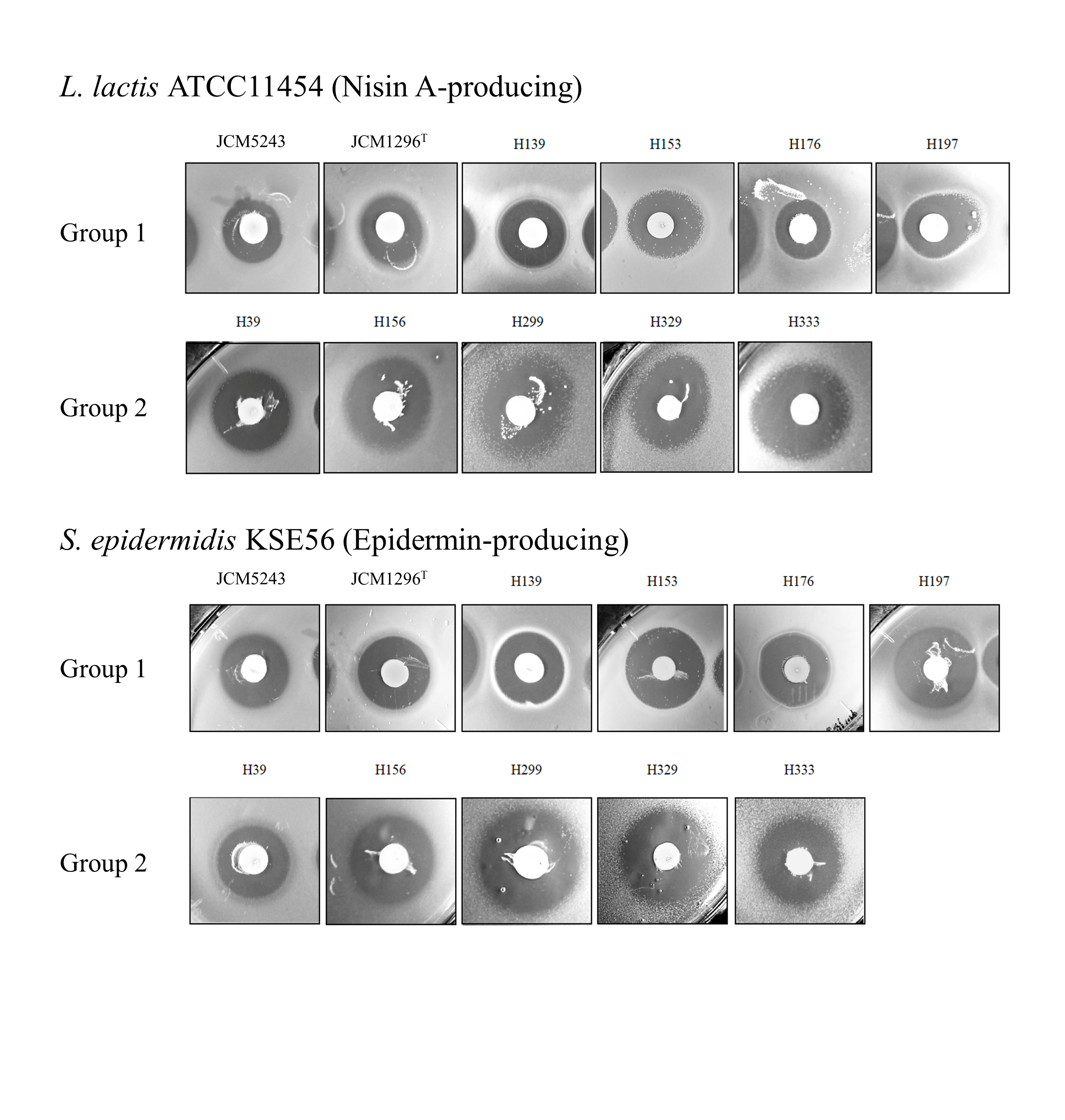

Supplement: S1 Fig — Direct assay was performed to evaluate the susceptibility to nisin A and epidermin. (TIF) [file pone.0280676.s001.tif]

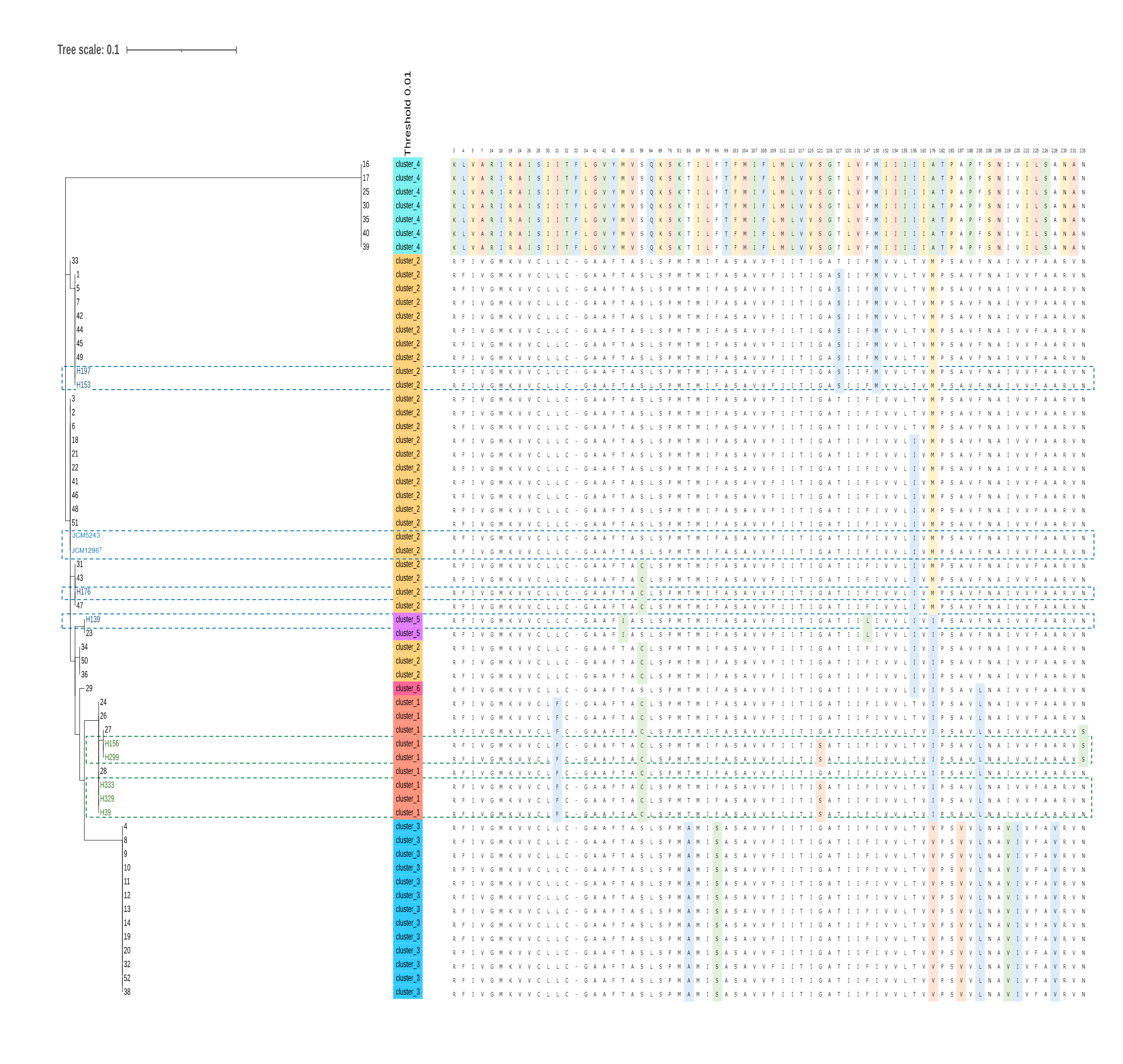

Supplement: S2 Fig — A phylogenetic tree was constructed with 11 the genome sequences determined in this study and 50 sequences obtained from the NCBI database. The blue-dashed square and green-dashed square show strains in Group 1 and Group 2, respectively. (TIF) [file pone.0280676.s002.tif]

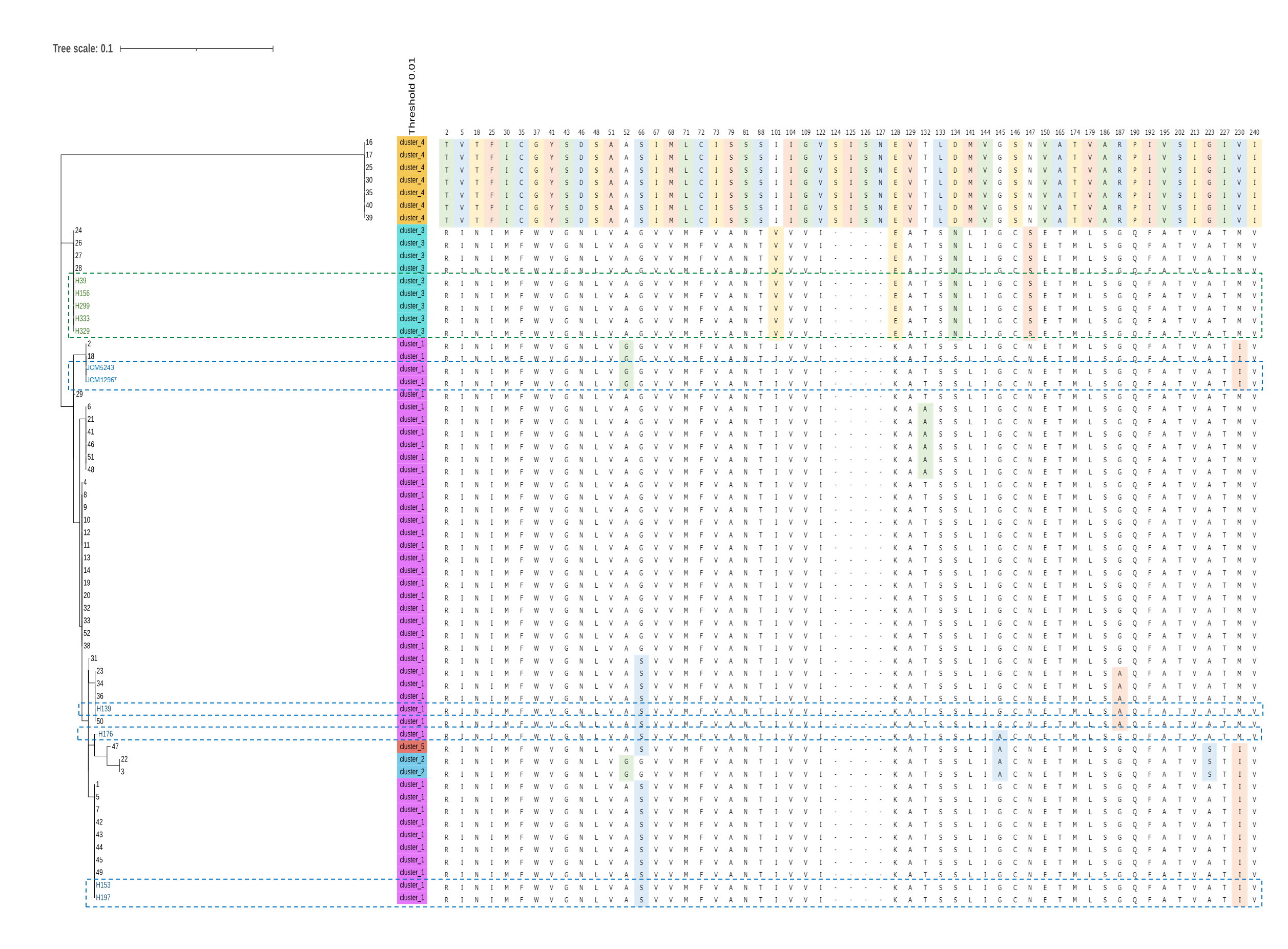

Supplement: S3 Fig — A phylogenetic tree was constructed with 11 the genome sequences determined in this study and 50 sequences obtained from the NCBI database. The blue-dashed square and green-dashed square show strains in Group 1 and Group 2, respectively. (TIF) [file pone.0280676.s003.tif]

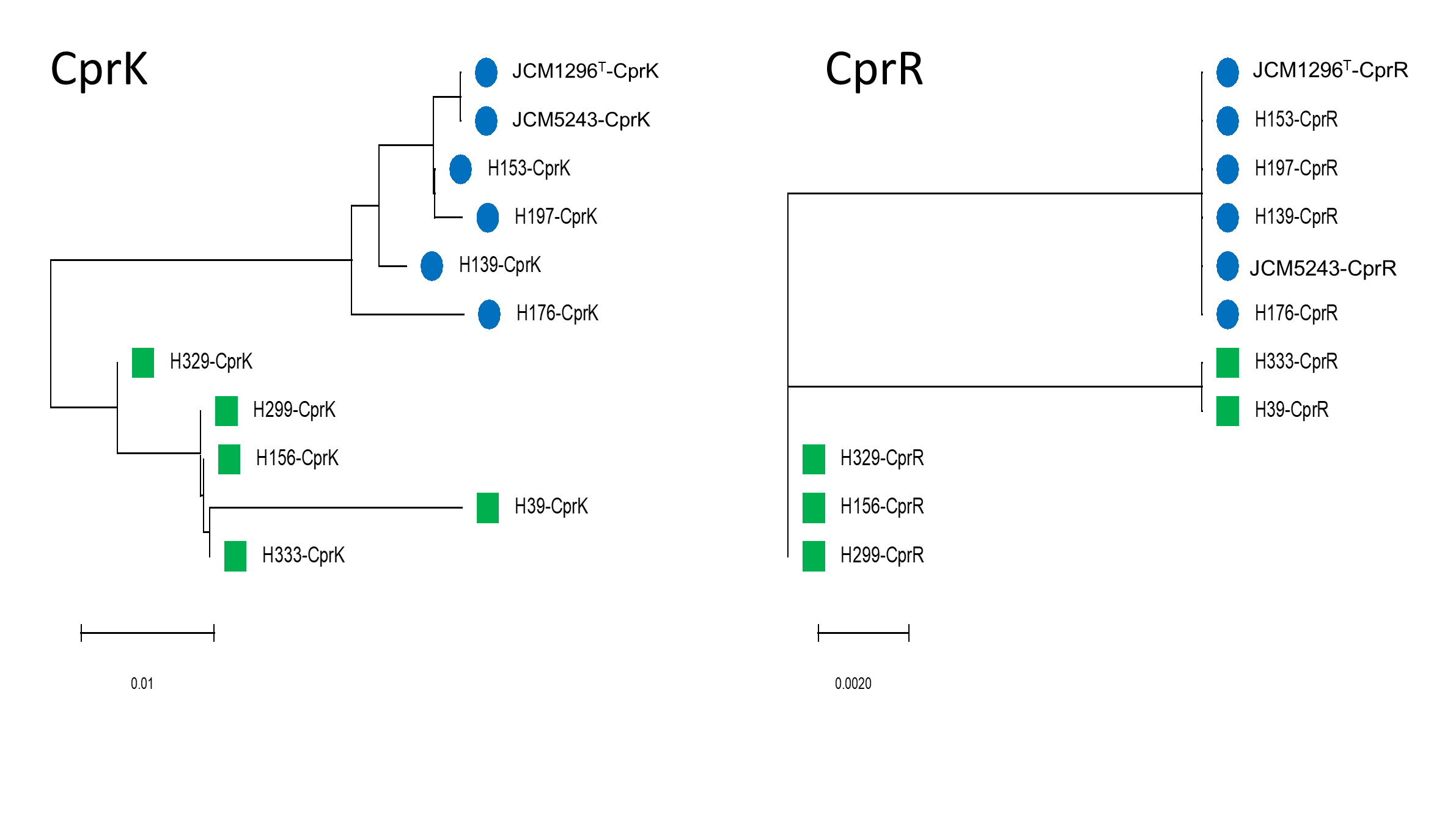

Supplement: S4 Fig — Phylogenetic analysis of CprK and CprR among 11 C. difficile strains. (TIF) [file pone.0280676.s004.tif]

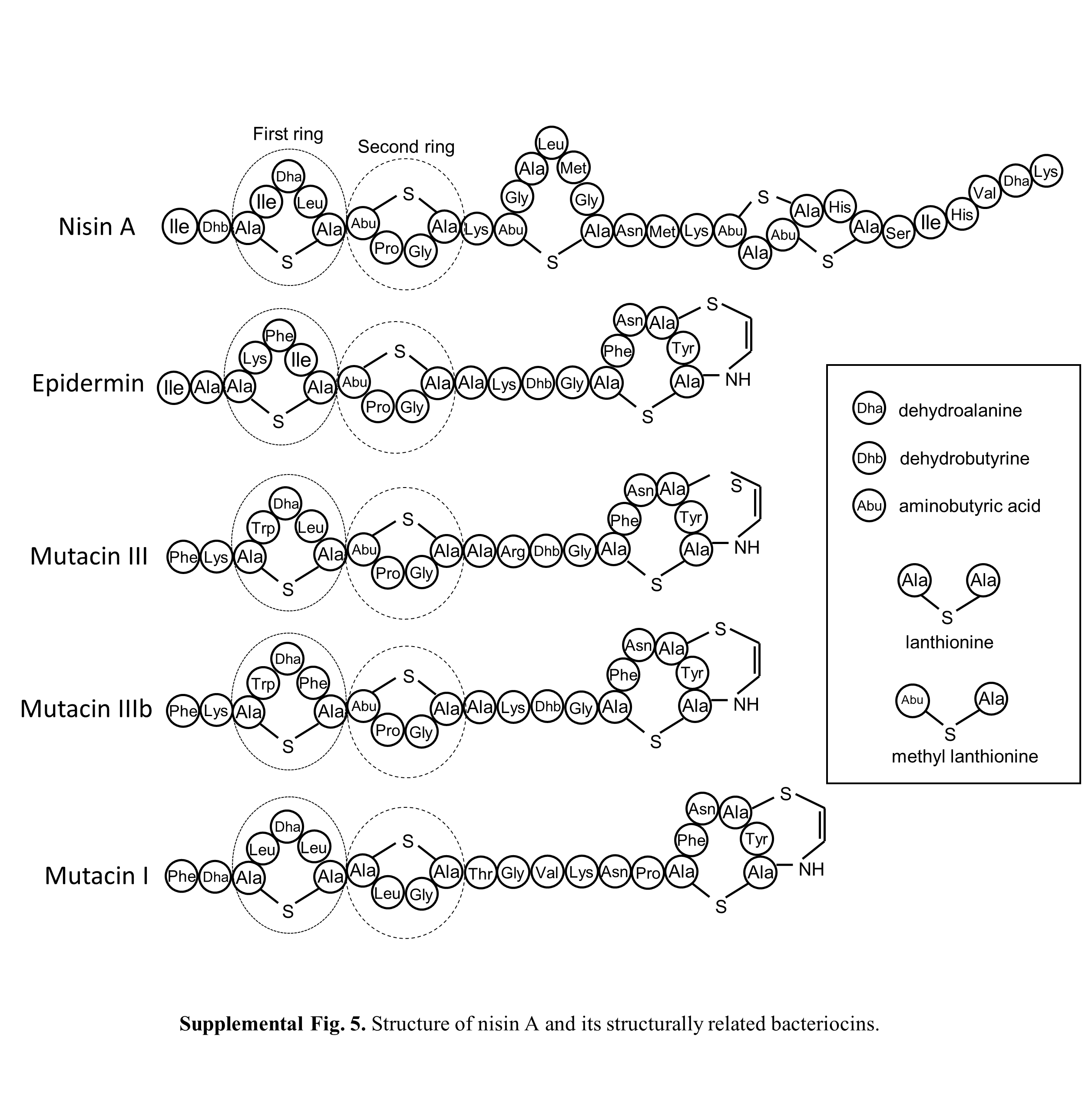

Supplement: S5 Fig — (TIF) [file pone.0280676.s005.tif]

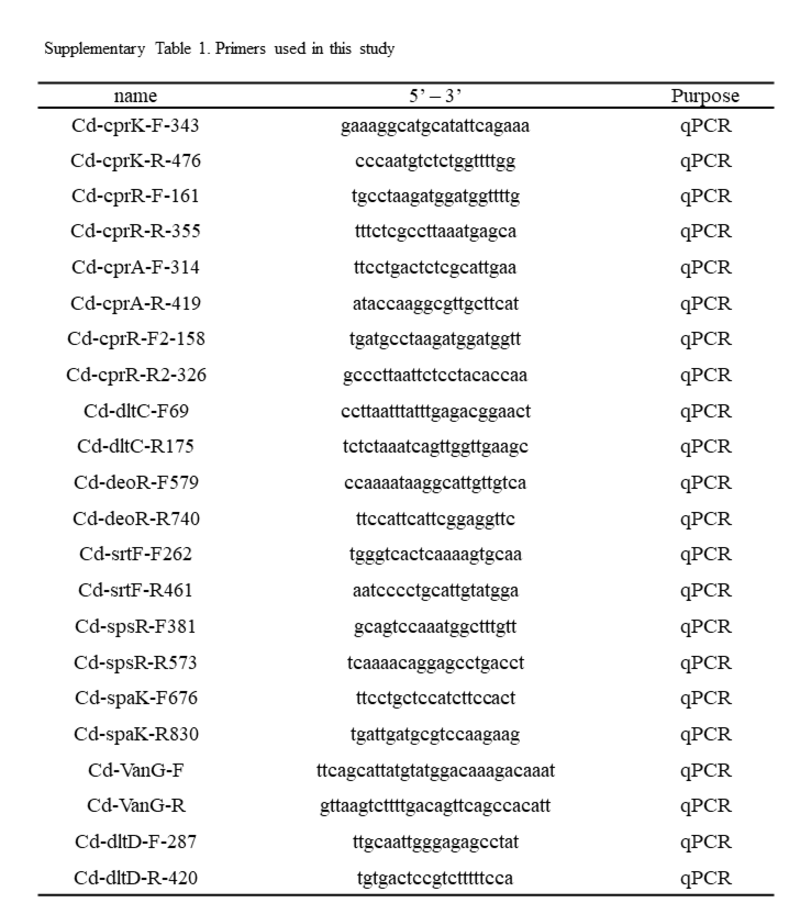

Supplement: S1 Table — (TIF) [file pone.0280676.s006.tif]
