# Supplementary material for: Different CprABC amino acid sequences affect nisin A susceptibility in Clostridioides difficile isolates
Source: PLoS One. 2023 Jan 20;18(1):e0280676. doi: 10.1371/journal.pone.0280676 (PMC9858009; doi:10.1371/journal.pone.0280676)
Supplement: Attachment — Submitted filename: Response to Reviewers.docx [file pone.0280676.s007.docx]

Dear Editor,

Thank you for your constructive comments. Based on the reviewer’s comments, we revised our manuscript considerably.

Reviewer #1: The work is well thought out, and carried out the related tests.

The perspectives that the author proposes are appropriate to the results found.

I have brief doubts, which I will write here considering the line number:

Line 18: replace gut microflora with gut microbiota

Our response: According to the suggestion, we revised it.

Line 121: include a “.” after “Table 1”

Our response: According to the suggestion, we revised it.

Line 125: Missing to include the country of Becton Dickinson and Company

Our response: According to the suggestion, we revised it.

Table 1. Why are the recent isolates written as Clostridium, considering that they are now called Clostridioides?

Our response: According to the suggestion, we revised it.

Line 15. Metronidazole should be lowercase in this case.

Our response: According to the suggestion, we deleted this sentence.

Line 176: mentions that qPCR, as previously indicated, however, does not include any reference.

Our response: According to the suggestion, we put the reference about qPCR method.

Line 182: what is the reason for only using the two indicated strains (JCM5243 and H39)?

Our response: According to the suggestion, we revised it to explain the reason.

Line 190: what was the maximum time before the use of the spores?

Our response: According to the suggestion, we revised it.

Line 225: remove spaces between Table 2 and throughout the text, and check the space between words.

Our response: According to the suggestion, we revised it.

Line 293: unify “Fig.” throughout the text; sometimes, it is not written the same.

Our response: According to the suggestion, we revised it throughout the text.

Table 4: Is there any parameter according to the CLSI to determine the multi-resistance or susceptibility of C. difficile?

Our Response: There are MIC breakpoints of several antibiotics according to CLSI. However, among antibiotics used in this study, vancomycin and metronidazole are included in the MIC breakpoints of *C. difficile* by CLSI, but other antibiotics are not included. However, we wanted to compare the MIC value between 2 groups and found no correlation between 2 groups.

Line 313: from here on, there are values between “( )” bibliographical references that clarify and homogenize the text.

Our response: According to the suggestion, we revised it.

Line 450: replace flora with microbiota

Our response: According to the suggestion, we revised it.

Line 515. Homogenize references; here, the year appears in parentheses, and in the others it does not.

Our response: According to the suggestion, we revised it.

Reviewer #2: In recent years, the problem of drug-resistant bacteria has been the most important issue in the therapeutic treatment of bacterial infections. In the case of Clostridium spp. infection, the occurrence of drug-resistant bacteria has also become serious problem, and the response to this problem needs to be addressed.　In this paper, using 11 clinical isolates of Clostridium difficile, the authors examined the susceptibility to several bacteriocins, including nisin A, and antibiotics. The results of the nisin A susceptibility test showed that the isolates were classified into two major groups, and the classification was similar for the other bacteriocin susceptibility except Mutacin IV. Additionally, no correlation was found between the expression levels of cpr or dlt genes, a group of molecules involved in nisin A resistance, and its susceptibility. Thus, the authors performed a comparative analysis of the whole genome sequences among the isolates to determine the cause of the differences in nisin A susceptibility. As results, the exact same two group formations seen in the susceptibility were confirmed by the genetic information analysis. Furthermore, the authors found that the amino acid sequences of Cpr molecules are unique to each group of bacteria, and speculated that the differences in the amino acid sequences affects the susceptibility to nisinA.

The reviewer considers the current manuscript to be well organized and sufficient for a publication of PLOS ONE if the author will provide suitable responses to the following minor comments;

1.The authors test susceptibility to several bacteriocins in the manuscript. In most bacteriocin susceptibilities, there is a significant difference between the two groups formed by nisinA susceptibility, but not in one. It is strongly expected that this may be caused by structural features of the bacteriocins. However, the current manuscript lacks information on the structure of bacteriocins and their structure-activity relationships. The reviewer recommend that the authors provide a figure showing bacteriocin structure in this regard.

Our responses: According to the suggestion, we showed the structure of bacteriocins including nisin A and its structural related bacteriocins which were discussed in Discussion section as the supplemental figure (Supplemental Fig. 5).

2. Related to comment #1.

What would happen to the susceptibility of isolates if other bacteriocins with less structural homology to nisinA, as like mutacin IV, were used in the test? If the authors have any information on this point, they should provide it or discuss it.

Our responses: According to the suggestion, we investigated the susceptibility of other bacteriocins with no structural relation to nisin A. As the result, we found no tendency as nisin A susceptibility. Therefore, the different amino acid sequences of CprA-C only affected the susceptibility of nisin A and its-related bacteriocins. We revised Table 3 and Fig. 3 by adding two bacteriocins, and also discussed it in Discussion section.

3. The pathogenicity of C. difficile has been well studied, and the typical toxins Tcds and CDT are known to be closely related to its virulence. In this study, the authors have divided the isolates into two groups according to nisinA susceptibility, are there any commonalities in the virulence factors in each group? The authors could add an explanation on this point based on whole genome information, which would further strengthen the value of this paper.

Our responses: We investigated the genes coding for Cdt and Txoin AB (TcdAB), and found a tendency of *cdt* genes in Group I and II, but did not find the tendency of *tcdAB*. We added these results in Discussion section.

4. The authors need to provide more detailed information regarding the source of the bacteria used in this paper.

Our responses: According to the suggestion, we added the detailed information of the strains in Methods section. Also, the strain name of JCM1296 was corrected to JCM1296^T^.

5. The authors use taurocholate in the experiments. The purpose of its use should be stated in the Methods section.

Our responses: According to the suggestion, we describe the purpose of taurocholate in Material and Methods section.

6. (Line121) Add period after “Table 1”.

Our responses: According to the suggestion, we revised it.

7. (Line176) The authors should add a reference paper regarding qPCR experiment.

Our responses: According to the suggestion, we put the reference.
